# Facilitation as Attenuating of Environmental Stress among Structured Microbial Populations

**DOI:** 10.1155/2016/5713939

**Published:** 2016-01-21

**Authors:** Suzana Cláudia Silveira Martins, Sandra Tédde Santaella, Claudia Miranda Martins, Rogério Parentoni Martins

**Affiliations:** ^1^Laboratory of Environmental Microbiology, Department of Biology, Sciences Center, Federal University of Ceará, Pici Campus, Block 909, 60455-760 Fortaleza, CE, Brazil; ^2^Sea Sciences Institute, Federal University of Ceará, 60165-081 Fortaleza, CE, Brazil; ^3^Graduate Program of Ecology and Natural Resources, Department of Biology, Sciences Center, Federal University of Ceará, 60455-760 Fortaleza, CE, Brazil

## Abstract

There is currently an intense debate in microbial societies on whether evolution in complex communities is driven by competition or cooperation. Since Darwin, competition for scarce food resources has been considered the main ecological interaction shaping population dynamics and community structure both in vivo and in vitro. However, facilitation may be widespread across several animal and plant species. This could also be true in microbial strains growing under environmental stress. Pure and mixed strains of* Serratia marcescens* and* Candida rugosa* were grown in mineral culture media containing phenol. Growth rates were estimated as the angular coefficients computed from linearized growth curves. Fitness index was estimated as the quotient between growth rates computed for lineages grown in isolation and in mixed cultures. The growth rates were significantly higher in associated cultures than in pure cultures and fitness index was greater than 1 for both microbial species showing that the interaction between* Serratia marcescens* and* Candida rugosa* yielded more efficient phenol utilization by both lineages. This result corroborates the hypothesis that facilitation between microbial strains can increase their fitness and performance in environmental bioremediation.

## 1. Introduction

The vision of a natural world dominated by conflict and deprivation has prevailed since Darwin (1858) and has been reinforced by contemporary ecologists [1]. However, in the last 30 years experiments have demonstrated the effect of facilitation on population distributions [2], community dynamics [3], species composition [4], and individual fitness [5].

Examples of facilitation between species are most familiar in the natural macroscopic world [6] but are much less explored in microbial communities especially because of the complexity of microbial interactions, which complicates data collection [7]. However, Morris et al. [8] described facilitation of a marine unicellular cyanobacterium of the* Prochlorococcus* genus when cocultured with heterotrophic marine bacteria and Pekkonen and Laakso [7] reported facilitative interactions between* Serratia marcescens* and* Novosphingobium capsulatum*.

Biofilms are spatially structured communities of microorganisms attached to surfaces or associated with interfaces. Constitute the dominant form in nature [6] and for this reason, in this study, the microbial cells were immobilized as biofilm on polyurethane foam coupons.

A central idea of Darwin's thesis is that organisms vary in their ability to leave descendants, a trait that is now generally called “Darwinian fitness” or simply “fitness” [9]. Although it is generally recognized that fitness is determined by the complete survival and reproductive schedules of individual organisms, experimental studies have rarely attempted to integrate these into a single measure of individual fitness [10].

The idea that facilitative interactions increase as environmental conditions become more stressful has become a ruling paradigm in ecology [11]. In ecological and evolutionary terms, stress is any extrinsic influence that reduces the Darwinian fitness [11]. These influences can be natural (temperature, salinity, winds, currents, and waves) or anthropic (pollution by toxic and recalcitrant chemicals) [3].

Under the hypothesis that the coexistence of structured microbial populations under stress increases their fitness, we predicted that the fitness index (FI) of microbial strains in consortium should be greater than the individual culture. As phenol is highly toxic to microorganisms, even at low concentrations (e.g., 10 mg L^−1^), and it can be found in most industrial effluents [12], this compound was used in this study as a model to represent chemical stress on microbial species. For the purpose of this study, the specific cell growth rate (*μ*) is defined as the fitness of microbial populations and the ratio of *μ* of a microbial strain under stress, in consortium and as an individual culture, is expressed by a fitness index (FI).

The microbial species chosen for the present experiment were* Serratia marcescens* and* Candida rugosa*, respectively, a Gram-negative, rod, facultatively anaerobic bacterium belonging to the family* Enterobacteriaceae* [13] and nonsporogenic, pseudofilamentous, unicellular, and nonpathogenic yeast [14]. These species were chosen for the fact that (i) they originate from environments impacted by toxic chemical pollutants, (ii) they can be cultivated both in isolation and together in laboratory conditions, (iii) they are easily separated based on colony morphology, as* S. marcescens* forms red colonies whereas* C. rugosa* forms cream colonies when grown on nutrient agar plates, and (iv) they coexist and grow on variety of substrates and according to Martins et al. [15] they are capable of adhering to polyurethane foam and create biofilms.

## 2. Material and Methods

### 2.1. Study Plan

A batch culture experiment was conducted to test how phenol affects growth rates (fitness) of the bacterium* Serratia marcescens* and yeast* Candida rugosa* previously immobilized on polyurethane foam coupons, in individual cultures and in microbial consortium. Minimum mineral medium containing different concentrations of phenol was inoculated with either* S. marcescens* or* C. rugosa* and with both species.

Pure and mixed immobilized strains of* Serratia marcescens* and* Candida rugosa* were grown in mineral culture media containing phenol. Population size in batch cultures with cells immobilized on solid polyurethane foam supports was measured as the number of living cells in intervals of 24 hours during 72 h and the growth rates of species in individual and microbial consortium were measured based on changes in the viable cell counting. Growth rates were estimated as the angular coefficients computed from linearized growth curves. Fitness index was estimated as the quotient between growth rates computed for lineages grown in isolation and in mixed cultures.

### 2.2. Microorganisms

*Serratia marcescens* was isolated from a treated effluent sample from the Effluent Treatment Plant at Pici Campus, Federal University of Ceará (ETE-PICI), Fortaleza, CE, Brazil, before chlorination, later identified and characterized according to Holt et al. [13].* Candida rugosa* was isolated from an untreated effluent sample from the company Lubrificantes e Derivados de Petróleo do Nordeste (LUBNOR), also in Fortaleza, and was identified according to Rocha et al. [16].

*Serratia marcescens* was preserved in deep tubes of nutrient agar (NA) (Merck, Germany) and* Candida rugosa* in deep tubes of potato dextrose agar (PDA) (Merck, Germany) both covered with mineral oil, at 4°C.

### 2.3. Preparation and Standardization of the Inoculum

Each strain was separately inoculated onto Petri dishes with a Luria-Bertani (LB) (Difco) medium containing (per liter) 10 g of tryptone, 5 g of yeast extract, 5 g of NaCl, and 17 g of agar. The plates were incubated at 30°C for 48 h (bacterium) and 72 h (yeast). Then, one colony of each strain was transferred to 100 mL of LB broth and incubated for 18 h at 26°C on a horizontal orbital shaker (150 rpm). Afterwards, 1 mL of each culture was transferred to 100 mL of LB broth and incubated again under the same conditions [17]. The optical density (OD) of each suspension was adjusted to approximately 0.6 at 600 nm, corresponding to between 10^8^ and 10^9^ CFU mL^−1^ estimated in a standard curve between the OD and the cell concentration (CFU mL^−1^) values. These curves were previously plotted for* S. marcescens* and* C. rugosa*.

### 2.4. Material for Cell Immobilization

Polyurethane foam of density 23 kg m^−3^ was cut into coupons of dimensions of 10 × 10 × 5 mm, corresponding to a surface area of 4.0 cm^2^. The coupons were washed with liquid detergent and water, rinsed with distilled water, immersed in 70% ethyl alcohol for 1 h, rinsed with distilled water, dried at 70°C for 4 h, sterilized at 121°C for 15 min, and dried overnight at 70°C [15].

### 2.5. Immobilization of Individual Cultures and Microbial Consortium on Polyurethane Foam Coupons 

A volume of 100 *μ*L of each strain in individual culture and 50 *μ*L of each species (1 : 1) in microbial consortium was transferred to 500 mL Erlenmeyer flasks containing 250 mL of LB broth (pH 5.5). Polyurethane foam coupons were added to each Erlenmeyer flask, to partially fill the air-liquid interface. The flasks were incubated at 26°C in a horizontal orbital shaker (150 rpm) for 24 h (bacterium) and 48 h (yeast). After cultivation, the polyurethane foam coupons were washed three times with distilled water to remove the unattached cells and dried at room temperature for 30 min.

Cell concentration of individual culture and microbial consortium was monitored by counting viable cells at time zero and every 2 h, until cell concentration was constant, which is typical of the stationary growth phase. The experiment was replicated twice and the viable cell count (CFU cm^−2^) for each experiment was performed in triplicate.

### 2.6. Quantification of Cells on Polyurethane Foam Coupons of Individual and Microbial Consortium

The number of cells on polyurethane foam coupons of individual and microbial consortium was determined by the plate count method tested and recommended by Parizzi et al. [18] for enumeration of bacterial adherence to inert surfaces as described below.

At the incubation times previously specified, four coupons were removed at random from each Erlenmeyer flask containing cells grown separately and consociated and were added to test tubes (12 × 120 mm) with 10 mL of peptone water diluent at 0.1% (w/v). These tubes were shaken on a vortex mixer for 1 min to release the weakly adherent cells. This suspension was discarded. The cells that remained on the coupons after this procedure were considered adherent and the tubes were shaken on a vortex mixer for 1 min.* Serratia marcescens* suspensions were diluted and inoculated onto plates containing plate count agar (PCA) (Merck, Germany) and incubated at 37°C for 48 h. Yeast cell counting was carried out in a PDA medium modified by the addition of tartaric acid to pH 3.5 and incubated at 25°C for 72 h [19]. In microbial consortium colony morphology was observed to differentiate yeasts from bacteria.

Viable cell counts of individual cultures and the microbial consortium on polyurethane foam coupons were carried out in triplicate. The results were expressed as colony forming units per cm^2^ (CFU cm^−2^) according to the equation CFU cm^−2^ =  *V*(*a*/*A*) × *M* × *D*, where *V* is the volume of diluent (mL); *a* is the plated aliquot (mL); *M* is the average cell count after incubation (CFU mL^−1^); *D* is the decimal dilution; and *A* is the coupon area (cm^2^) [20].

### 2.7. Fitness of Microbial Strains

A phenol stock solution was prepared by dissolving 2 g of phenol (purity higher than 99%, Sigma-Aldrich) in 1000 mL of distilled water. This solution was sterilized separately by filtration using 0.22 *μ*m membranes (Millipore, USA).

The synthetic mineral medium (SMM) (mg L^−1^) (NH_4_SO_4_ 2000, NaNO_3_ 1000, KH_2_PO_4_ 200, MgSO_4_ 250, CaCl_2_·2H_2_O 80, CuSO_4_·7H_2_O 50, H_2_MoO_4_ 50, MnSO_4_·7H_2_O 50, Fe_2_ (SO_4_)_3_ 50, and ZnSO_4_ 40) [21] was distributed in 250 mL Erlenmeyer flasks (100 mL each) and sterilized at 121°C for 15 min. The flasks were cooled to room temperature (26°C) and phenol from the stock solution at different concentrations was added to obtain final concentrations of 62.5, 125, 250, 500, and 1000 mg L^−1^. The final pH was 5.5.

For the construction of growth curves 70 polyurethane foam coupons containing cells from the individual and consortium were inoculated into 250 mL Erlenmeyer flasks containing 100 mL of MMS with phenol at concentrations of 62.5 mg L^−1^, 125 mg L^−1^, 250 mg L^−1^, 500 mg L^−1^, and 1000 mg L^−1^. The flasks were incubated under aerobic conditions on a rotary shaker (150 rpm) at 26°C for 72 h. Four coupons were collected at random from each phenol concentration every 24 h. For each dilution of phenol the experiment was replicated twice and the viable cell count for each repetition was performed in triplicate. The results were expressed as CFU cm^−2^.

The fitness index (FI) was used to determine the predominant type of interactions and was calculated as the ratio of the exponential growth rates of a microbial strain under stress, in consortium versus individual culture. FI values >1 were considered positive interactions; <1 as negative interactions; =1 as neutral interaction.

### 2.8. Statistical Analyses

The linearity of each strain in individual culture and microbial consortium was determined to establish the FI. Thus, incubation times (*x*-axis, time in h) were plotted against log-transformed cell concentrations (*y*-axis, log_10_ CFU cm^−2^) for each microbial species, at the respective phenol concentrations. Least squares regression was performed using GraphPad Prism 5.00 (GraphPad Software, San Diego, CA). The intercept and slope of the straight line were obtained by adjusting the data to a linear model (represented by the equation *y* = *ax* + *b*, where *b* is the *y* intercept and *a* is the slope or angular coefficient). The suitability of the model fit (linearity) was expressed as the correlation coefficient (*r*). The slope of the regression line, with 95% confidence intervals (95% CI), was also calculated to determine the statistical significance of the slope. The specific growth rates were determined by using the angular coefficient of the linear regression (*a*) calculated for the period corresponding to the exponential phase of culture growth [22].

The *t*-test was used to compare slopes of the regression lines between individual cultures and microbial consortium of cells on polyurethane foam coupons. The paired *t*-test was used to compare cell concentration of individual cultures and microbial consortium at each phenol concentration.

## 3. Results and Discussion

**Figure 1:**
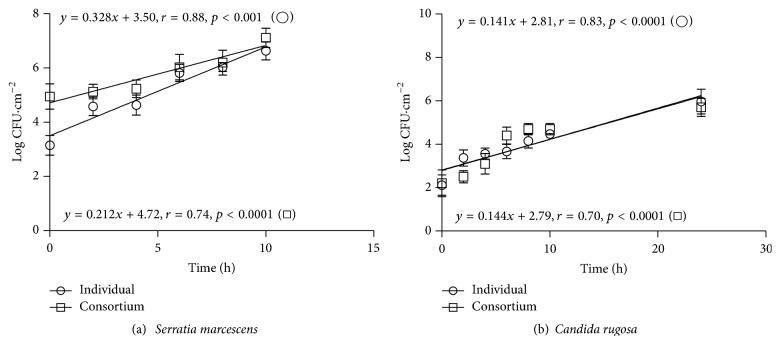
Growth curves of* Serratia marcescens* (a) and* Candida rugosa* (b) as individual cultures (○) and in a microbial consortium (□) on polyurethane foam coupons immersed in the Luria-Bertani (LB) broth for 24 h.

The linear regression equations for* Serratia marcescens* as individual culture and in consortium with* Candida rugosa* on polyurethane foam coupons (structured environment) is represented in Figures 1(a) and 1(b) where *a* is a constant representing the slope of the line and *b* a constant representing the intercept of the straight line with the vertical axis.

A positive (*r* > 0.80) and significant linear relationship (*p* < 0.05) was observed between bacteria concentration and incubation time in individual and consortium cultures (Figure 1(a)). Although* S. marcescens* was more prevalent compared with* C. rugosa*, the angular coefficient of the curves (0.328 and 0.212), which represent* S. marcescens* specific growth rates (*μ*), when in individual cultures and in consortium with* C. rugosa*, were not significantly different (*p* > 0.05), thus indicating that the yeast presence did not affect the bacterial growth rate (Figure 1(a)).

A positive (*r* > 0.80) and significant linear (*p* < 0.05) relationship between cell concentration and time was also observed for* C. rugosa*, individually and in consortium with* S. marcescens* (Figure 1(b)). The specific growth rates of* C. rugosa* in individual and consortium cultures were virtually the same (0.141 and 0.144, resp.), indicating that the bacterium did not affect the yeast growth rate (Figure 1(b)).

The predominance of* S. marcescens* in consortium with* C. rugosa* grown on polyurethane foam coupons may be related to the greater potential for initial adhesion, as observed by Martins et al. [15]. The specific growth rate of* S. marcescens*, in individual and consortium cultures, was significantly higher than* C. rugosa*, even under pH and incubation temperature more favorable to the yeast. However, the yeast continued to grow even after the bacterium ceased. This result confirms that, in general, bacterial cells have a faster growth rate than yeast cells due to their higher area : volume ratio [23].

Experiments with bacteria and yeasts grown immobilized in consortium are scarce, but Abe et al. [24] did not find significant differences between the yeast* Saccharomyces cerevisiae* BY4741 and the bacterium* Lactobacillus plantarum* HM23 when grown in individual and consortium biofilm cultures, concluding that no mutual influence occurred between the species. Due to the risk of human infection by pathogenic biofilms the effect of synergistic interactions between pathogenic bacteria and yeasts has been described for venous and urinary catheters, heart valves, and dental materials [25]. Regarding the last substrate, the association between* Streptococcus mutans* and* Candida albicans* improved the adhesion capacity of both species [26]. In vitro studies of clinical biofilms showed that* Candida albicans* increased the growth rate of* Staphylococcus epidermidis* and* Staphylococcus aureus* [27].

**Figure 2:**
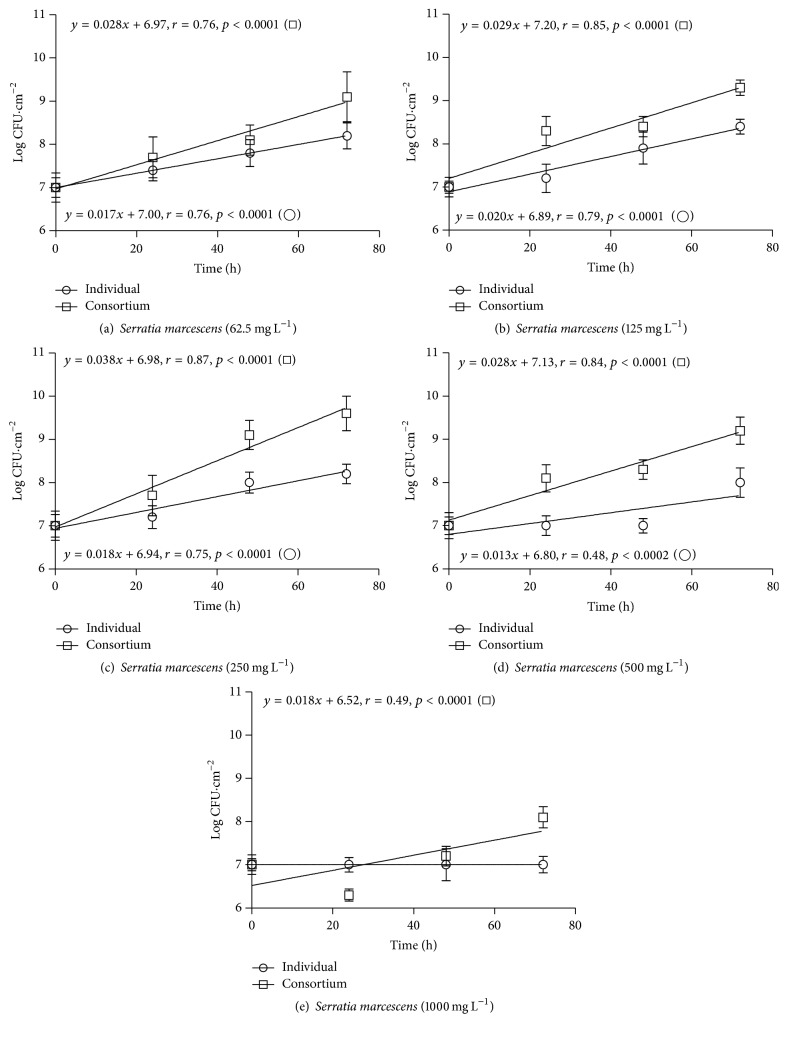
Growth curves of* Serratia marcescens* on polyurethane foam, as an individual culture (○) and in a consortium with* Candida rugosa* (□), at phenol concentrations of (a) 62.5 mg L^−1^ (b) 125 mg L^−1^, (c) 250 mg L^−1^, (d) 500 mg L^−1^, and (e) 1000 mg L^−1^.

**Figure 3:**
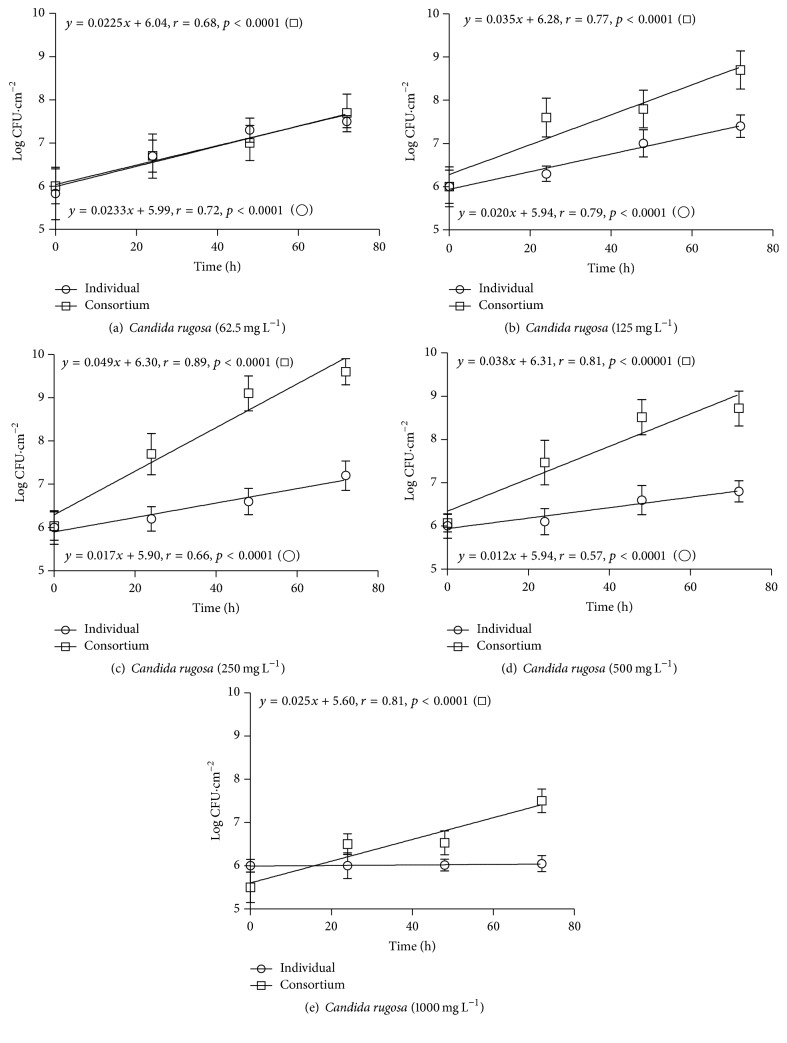
Growth curves of* Candida rugosa* on polyurethane foam, as an individual culture (○) and in a consortium with* Serratia marcescens* (□), at phenol concentrations of (a) 62.5 mg L^−1^, (b) 125 mg L^−1^, (c) 250 mg L^−1^, (d) 500 mg L^−1^, and (e) 1000 mg L^−1^.

The linear regression equations for* S. marcescens* and* C. rugosa* strains (*y* = *ax* + *b*), grown on polyurethane foam coupons, as individual and consortium cultures, and under different phenol concentrations, are presented with the respective growth curves in Figures 2(a), 2(b), 2(c), 2(d), and 2(e) and Figures 3(a), 3(b), 3(c), 3(d), and 3(e).

As a monoculture, the bacteria exhibited a positive (*r* > 0.70) and linear relationship (*p* < 0.05) between cell concentration and contact time at phenol concentrations of 62.5 mg L^−1^, 125 mg L^−1^, and 250 mg L^−1^ (Figures 2(a), 2(b), and 2(c)), for 500 mg L^−1^ (*r* = 0.48) (Figure 2(d)). Bacterial growth was inhibited at 1000 mg L^−1^ of phenol (Figure 2(e)).

In the microbial consortium,* S. marcescens* showed better performance, as indicated by the positive (*r* > 0.80) and linear relationship (*p* < 0.05) with time, at phenol concentrations of 62.5 mg L^−1^, 125 mg L^−1^, 250 mg L^−1^, and 500 mg L^−1^, Figures 2(a), 2(b), 2(c), and 2(d), for 1000 mg L^−1^ (*r* = 0.49) (Figure 2(e)).

As an individual culture,* C. rugosa* also showed a positive (*r* > 0.65) linear relationship (*p* < 0.05) between cell concentration and contact time, at phenol concentrations of 62.5 mg L^−1^, 125 mg L^−1^, 250 mg L^−1^, and 500 mg L^−1^ (Figures 3(a), 3(b), 3(c), and 3(d)). At 1000 mg L^−1^ of phenol the growth was inhibited (Figure 3(e)). At 62.5 mg L^−1^ phenol no significant differences were observed (*p* > 0.05) between the regression coefficients of* C. rugosa* as an individual culture and in consortium with* S. marcescens*. However,* C. rugosa* had significantly higher (*p* < 0.05) regression coefficients when in consortium with* S. marcescens*, at phenol concentrations of 125 mg L^−1^, 250 mg L^−1^, and 500 mg L^−1^ (Figures 3(a), 3(b), 3(c), and 3(d)). At 1000 mg L^−1^ the growth of the yeast was inhibited as individual culture, but in consortium it presented a regression coefficient of 0.025 (Figure 3(e)).

The growth of* Serratia marcescens* and* Candida rugosa* at phenol concentrations of 62.5 mg L^−1^, 125 mg L^−1^, and 250 mg L^−1^ may be related to the origin of the species:* C. rugosa* from a petrochemical effluent containing phenol as one of the chemical pollutants [16] and* S. marcescens* from a wastewater treatment station that receives inputs of chemical pollutants from different research laboratories.

In the present study, the high microbial toxicity of phenol allows considering that dead cells could be used as nutrients by surviving cells, resulting in balance between live and dead cells, in phase called long-term stationary by Finkel [28]. However, since the only carbon and energy source in the minimal medium is phenol, the constant increase in the number of* Serratia marcescens* and* Candida rugosa* cells in individual and consociated culture showed phenol-degrading activity.

The types of interactions between microorganisms is based on the effect that one species has on the population size of the species with which it interacts [29]. In the present study, the significant increase in the populations of both microbial species when grown in consortium, under the effect of all the phenol concentrations, indicates mutualism was the predominant relationship.

For* C. rugosa* the fitness index (FI) showed values above 1 at phenol concentrations of 125 mg L^−1^, 250 mg L^−1^, and 500 mg L^−1^. At 62.5 mg L^−1^ FI was approximately 1. For* S. marcescens* the FI showed values above 1, for phenol concentrations of 62,5 mg L^−1^, 125 mg L^−1^, 250 mg L^−1^, and 500 mg L^−1^.

The individual growth inhibition at phenol concentration of 1000 mg L^−1^ for the bacterium and yeast could prevent the calculation of the relationship between the specific rates and consequently of the FI (Table 1).

Positive interactions between associations of microorganisms become more important with increasing stress intensity [30]. This fact was noted in the present study, since both strains had the highest fitness indexes at increasing phenol concentrations (Table 1).

We did not find similar studies of* C. rugosa* and* S. marcescens* in the literature, but in vitro the coexistence of the bacteria* S. marcescens* and* Novosphingobium capsulatum* in aquatic microcosms for 13 weeks showed predominance of positive interactions [7]. Ishii et al. [31] evaluated the possible interactions between the Archaea species* Pelotomaculum thermopropionicum* and* Methanothermobacter thermautotrophicus*, concluding that both species benefited from the interactions. A similar interaction between bacteria and Archaea species,* Thermotoga maritima* and* Methanococcus jannaschii*, was observed by Muralidharan et al. [32]. Rikhvanov et al. [33], studying the effect of* Bacillus* sp. on the growth of the yeast* Debaryomyces vanriji*, observed that* Bacillus* sp. strongly stimulated the growth of* D. vanriji* in mixed culture in liquid minimal medium at 30°C.

Studies of microbial consortia to increase the degradation rate of toxic compounds in industrial processes are indicators of the prevalence of cooperative interactions. In the presence of phenol, the bacteria* Propioniferax* PG-02 and* Comamonas* sp. PG-08 showed higher growth rates in a consortium than in individual cultures, according to Jiang et al. [34]. Jiang et al. [35] showed that the bacteria* Pandoraea* sp. PG-01 and* Rhodococcus erythropolis* PG-03 were functionally similar under the effect of phenol. Although* Pandoraea* sp. PG-01 was the strongest competitor, both strains grew in a consortium when phenol was the only carbon source. The biotechnological potential of* C. rugosa* and* S. marcescens* is evident. Although Hejazi et al. [36] described* S. marcescens* from clinical origin, as an emerging pathogen, many strains from environmental origin have been successfully evaluated for bioremediation application [37, 38]. The most studies on phenol biodegradation have used* Candida tropicalis* strains [39–41]; in this context, the* C. rugosa* strain of this study has potential for phenol biotransformation processes.

The study of microbial interactions is still at an early stage [42]; hence there is little consensus yet among authors. There are experimental data indicating that competitive interactions are predominant among microbial species [6, 43, 44]. Nevertheless, other studies that have documented positive interactions among microbial species [e.g., [45]] lead to conclusion that positive interactions are dominant in microbial communities. In this study, the positive interaction between* Serratia marcescens* and* Candida rugosa* under environmental stress was reflected in elevated growth rates of both organisms when cocultured at subinhibitory phenol concentrations. The result is an interesting addition to a growing number of experimental evidences on complex interactions within relatively simple microbial communities and provides additional evidence of the importance of cooperation among culturable microbial species. From the ecological perspective, the result of this study is a contribution to the debate on whether evolution in complex communities is driven by competition or cooperation.

## 4. Conclusions

Competitive interactions did not affect the fitness of either species in this study. The predominance of positive interactions in the consortium cultures, under chemical stress, in comparison with monocultures under the same stress condition corroborates the hypothesis that facilitation between microbial strains can increase their fitness and performance in environmental bioremediation.

## Figures and Tables

**Table 1:** Fitness index of *Serratia marcescens* and *Candida rugosa* under the effect of different phenol concentrations.

Phenol (mg L^−1^)	Fitness index (FI)	
	*Serratia marcescens*	*Candida rugosa*
62.5	1.65	0.96
125.0	1.45	1.70
250.0	2.10	3.00
500.0	2.00	3.20
1000.0	Indefinite	Indefinite

Positive interactions FI > 1, negative interactions FI < 1, and neutral interactions FI = 0.

The fitness index (FI) is the ratio of the exponential growth rates of each microbial strain in different phenol concentrations, in consortium and as individual culture.
